# Sustainable traditional grass cloth fiber dyeing using the Taguchi L16 (4^4) orthogonal design

**DOI:** 10.1038/s41598-022-18213-9

**Published:** 2022-08-16

**Authors:** Lina Lin, Tiancheng Jiang, Le Li, Md. Nahid Pervez, Cong Zhang, Chao Yan, Yingjie Cai, Vincenzo Naddeo

**Affiliations:** 1 Hubei Provincial Engineering Laboratory for Clean Production and High Value Utilization of Bio-Based Textile Materials, Wuhan Textile University, Wuhan, 430200 China; 2 Hubei Key Laboratory of Biomass Fibers and Eco-Dyeing & Finishing, Wuhan Textile University, Wuhan, 430200 China; 3 Sanitary Environmental Engineering Division (SEED), Department of Civil Engineering, University of Salerno, 84084 Fisciano, Italy

**Keywords:** Engineering, Materials science

## Abstract

For many centuries, traditional grass cloth has been used as an important raw material for home textiles in China, but its market can be expanded by incorporating color. Reactive Red 2 (R2), Reactive Blue 194 (B194), and Reactive Orange 5 (O5) were used in this work to explore the dyeing behavior of sustainable traditional grass fiber using industrial dyeing methods. Initially, an L16 (4^4) orthogonal design was schematically applied to carry out the dyeing process and it was determined that the total dye fixation rate (T%) of B194 dye was the best among the three dyes. Accordingly, a statistical Taguchi technique was analyzed on a larger scale to optimize the dyeing process parameters (salt concentration, fixation time, fixation temperature, and solution pH) of B194, in which solution pH was found to be the most influential factor in achieving the highest T%. This phenomenon was also verified using analysis of variance (ANOVA), where the solution pH was found to be the biggest contributor (50%) and statistically significant (p < 0.05). Finally, confirmation tests were conducted under optimized conditions and a higher T% (53.18%) was determined compared to initial conditions (48.40%). Later, Fourier transform infrared (FTIR) spectroscopy and scanning electron microscopy (SEM) were used to analyze the structural characteristics and found that grass cloth was chemically stable, yet gummy materials were still observed on their surface, which was also confirmed from digital photographs. Generally, the color coordinates and fastness properties were also satisfactory.

## Introduction

The traditional grass cloth, named Xiabu, can be traced back to more than 6000 years ago in China^1^, where it was primarily used in mourning clothes, crowns, hats and so on. The traditional grass cloth is artificially woven with gummy ramie yarns by hand, and the fabric exhibits a rough and stiff hand feel, good air permeability, and hydrophilic and antibacterial properties^2,3^. The traditional grass cloth has an important position in the development history of Chinese textiles because it embodies the lengthy history and aesthetic sensibility of the Chinese people. As a result, in 2008, the Chinese government has included this historical weaving method on the National Intangible Cultural Heritage List. The fast development of mechanized production has brought about significant changes in the traditional mode of production, and as a result, the home-based production of traditional grass cloth is facing unprecedented difficulties. At present, traditional grass cloth is still sold in markets for decorations, such as curtains, placemats, cushions, etc.^1^. Being able to generate a broad range of color shades of grass cloth is an excellent recommendation for marketing its goods and bringing awareness of the grass cloth tradition, since multicolored grass cloth is seldom seen on the market today^4^.

The grass cloth mainly consists of cellulosic fibers and gummy materials, including hemicellulose, pectin, and lignin^5,6^. Degumming is required for the production of fine ramie yarn, and various treatments such as chemical degumming, bio-degumming, and biochemically-combined degumming^7–9^ can be utilized based on the individual requirements of each ramie yarn process^10^. On the other hand, gummy materials provide a harsh and rigid performance^7^ and contribute to the unique texture of the traditional grass cloth, which are the crucial and most characteristic aspects of the traditional grass cloth. Besides, some modern tools such as ultraviolet irradiation^11^, ultrasound^12^, microwave irradiation^13^, gamma irradiation^14^, and plasma treatment^15^ can be applied to improve surface characteristics and dyeing performance. However, these tools are costly, environmentally unpleasant, and lack scale-up feasibility.

In our previous report, gummy ramie yarn was completely dyed with reactive dye, i.e., the gummy materials and cellulosic fibers formed covalent bonds with reactive dyes^16^. However, this process focused on the dye absorption and dispersion behaviors during dyeing with the addition of soda ash^17,18^. In reactive dyeing, the final color shade of the dyed substance is decided by the total dye efficiency (T%), which is based on the dye exhaustion percentage (E%) and dye fixation rate (F%)^19^. In general, dyeing conditions are influenced by various factors^20^ such as salt concentration, fixation time, fixation temperature, and pH of the dyebath, and it is necessary to develop a systematic approach to the planning, implementation, and evaluation of the process to generate the best results.

Traditionally, optimization procedures that maintain all parameters constant while making a single change are seen as time-consuming and costly. In this case, the design of experiments, often known as DOE, is a strategy that uses a systematic approach to identify the link between elements influencing a process and the output of that process. Generally, the DOE approach can be divided into two categories: full factorial design and Taguchi experimental design^21,22^. During full factorial design, all possible combinations of parameter values are evaluated and analyzed. Comparatively, only chosen levels are considered for evaluation in a Taguchi experimental design study. Taguchi method is considered a robust technique since it uses an orthogonal array (OA) design^23^. The OA can quantitatively identify the right parameters and levels and is used to decrease the number of trials, the duration of experiments, the cost, and the amount of human energy required. The Taguchi approach relies heavily on signal-to-noise (S/N) ratio and analysis of variance (ANOVA) tables to determine the statistical significance, which uses a response table to determine optimal conditions with the most influential factors. Confirmation tests have been subsequently used to verify the feasibility of experimental designs^24,25^. A number of researchers used Taguchi experimental design approach to improve the process quality of textile fiber dyeing.

Wahyudin et al.^26^ used an L9 (3^4) orthogonal array design in order to optimize the cotton knit fabric dyeing process, in which ANOVA analysis was the main statistical tool. It is confirmed from this study that the application of Taguchi plays an important role in reducing the re-dyeing process. Shafiq et al.^27^ showed that an L25 (5^4) Taguchi technique was feasible for extracting the natural dye under the optimized conditions and subsequently for the cotton fabric dyeing process. Hossain et al.^28^ used an orthogonal array-based Taguchi design of L9 (3^3) for deep dyeing of cotton fabric with cacao husk extract to maximize the exhaustion percentage. Therefore, the Taguchi technique was used to optimize the reactive dyeing process of grass cloth fiber, which, to the best of our knowledge, is the first research to be published on this subject.

This study explores the dyeing performance of sustainable traditional grass cloth fiber with three different kinds of reactive dyes, generally, low, medium, and high temperature categories, to determine which is the most effective. The Taguchi method was applied to analyze the influence of various dyeing factors on E%, F%, and T%, and finally the optimum dyeing conditions for each category of reactive dye were acquired, which is useful for the high efficiency dyeing of traditional grass cloth with reactive dye. Overall, the achievement of various colorful nature could be beneficial for tackling the challenge of coloration properties of the grass cloth fibers.

## Experimental

### Materials and dyeing process

**Figure 1:**
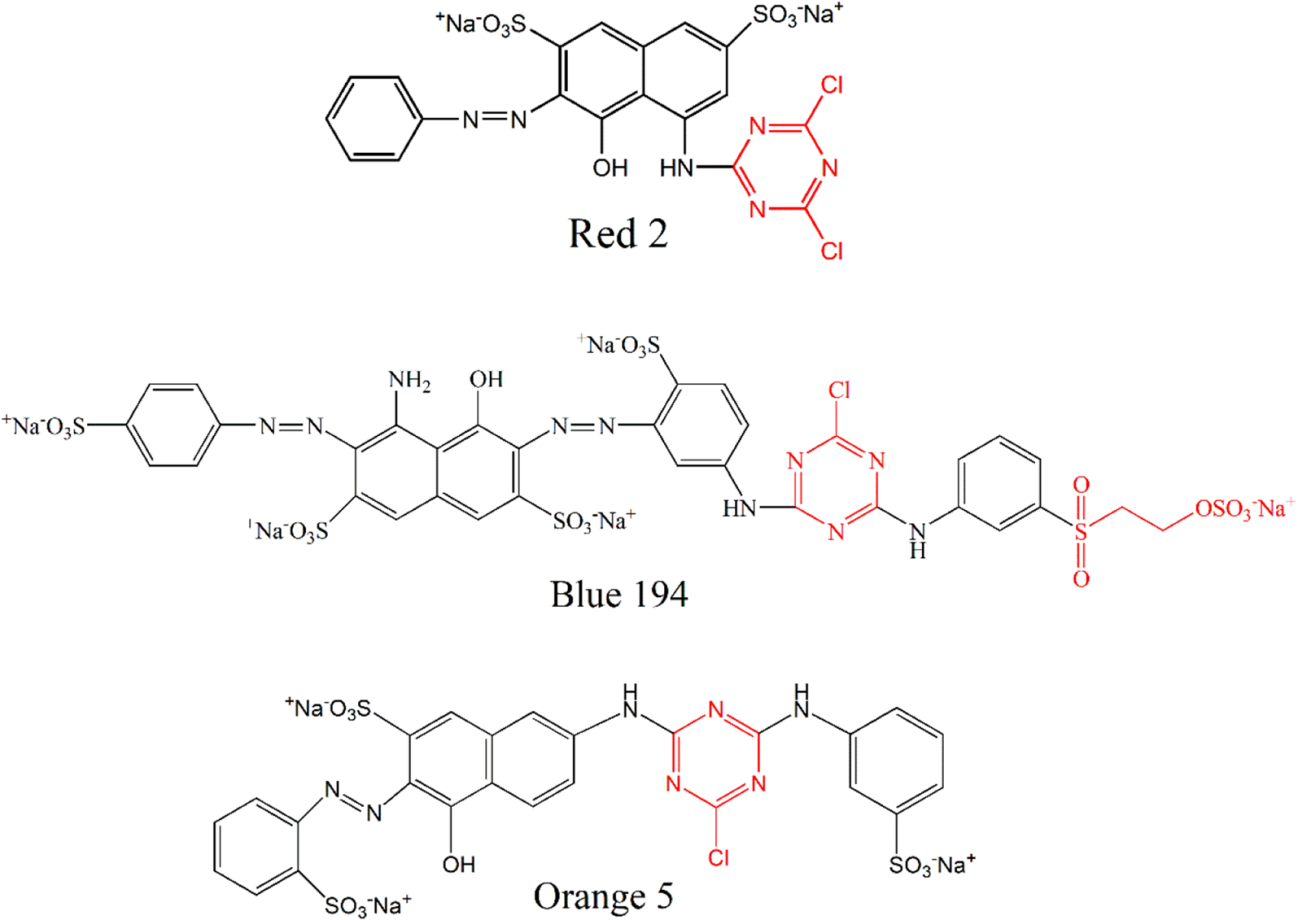
Molecular structures of the three dyes.

Traditional grass cloth (without degumming) was purchased from a local market. Reactive Red 2 (R2, low temperature type), Reactive Blue 194 (B194, middle temperature type), and Reactive Orange 5 (O5, high temperature type) were purchased from Shanghai Jiaying Chemical Company (China), and their molecular structures were shown in Fig. 1. Dalton UK Company (China) provided the non-ionic detergent (Luton 500) for the project. All other compounds were of analytical quality.

**Figure 2:**
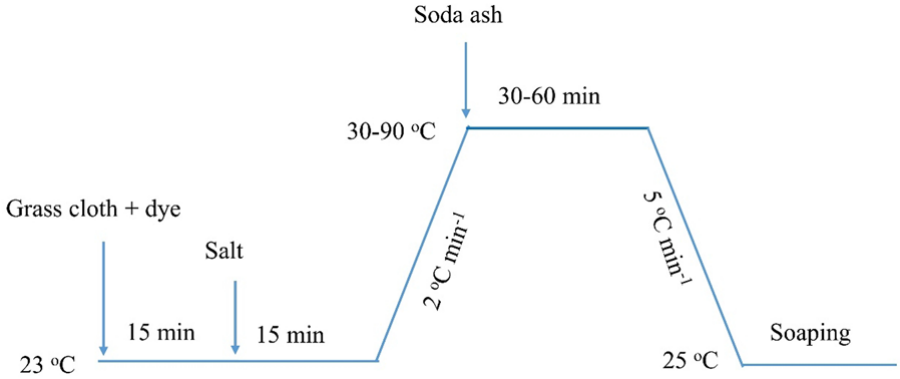
Dyeing process of grass cloth with R2, B194, and O5 in water.

For traditional grass cloth dyeings, 3% o.m.f (on mass of fiber) of reactive dye was employed at a liquor ratio of 50:1 with an automatic prototype rotary infrared radiation laboratory-dyeing apparatus (Model: A-12, AQUA, China). Other dyeing parameters are listed in Table 1, and the dyeing process in water is shown in Fig. 2. The salt was NaCl and the soda ash was Na_2_CO_3_ for pH 8–11 and NaOH for pH 12. The dyebath was heated to the desired temperature at a rate of 2 °C min^−1^ and maintained at the desired temperature for 30–60 min. After dyeing, a soap solution (2 g L^−1^ of non-ionic detergent) was supplied to wash the dyed samples at a liquor ratio of 50:1 at 95 °C for 15 min, accompanied by drying in an oven at 60 °C.

**Table 1:** L16 (4^4) orthogonal experimental scheme for exhaust dyeing.

Factor	Symbol	R2	B194	O5
Salt (g L^−1^)	A	50–80	50–80	50–80
Fixation time (min)	B	30–60	30–60	30–60
Fixation temperature (^o^C)	C	30–60	50–80	60–90
pH	D	8–11	8–11	9–12

### Measurement and characterization

E% is expressed as a percentage of dye mass that has been adsorbed onto a cloth after dyeing has taken place (Eq. 1)^29^ to the initial dye mass usage. E% was calculated according to Eq. (1), where A_0_ and A_1_ refer to the dye solution absorbances at its maximum adsorption wavelength (λ_max_) before and after dyeing, respectively. F% is the ratio of fixed dye in the material absorbed dye in that substance after dyeing. The soaping process washes off the unfixed dyes. Therefore, F% was computed using Eq. (2), where A_2_ is the absorbance of dye in the soap solution. T% is the rate of the fixed dye in the substance to the initial dye usage and is calculated by Eq. (3). An ultraviolet–visible spectrophotometer (Cary 300, Agilent Technologies, Australia) was used to measure the absorbance of the dye solution, and the λ_max_ values for R2, B194, and O5 are 540 nm, 600 nm, and 508 nm, respectively.

1 $$\text{E}\%=\frac{{\text{A}}_{0}-{\text{A}}_{1}}{{\text{A}}_{0}} \times {100}\%$$

2 $$\text{F}\%=\frac{{\text{A}}_{0}-{\text{A}}_{1}-{\text{A}}_{2}}{{\text{A}}_{0}-{\text{A}}_{1}} \times {100}\%$$

3 $$\text{T}\%=\frac{{\text{A}}_{0}-{\text{A}}_{1}-{\text{A}}_{2}}{{\text{A}}_{0}} \times {100}\%$$

To determine the color coordinates of the dyed sample, CIElab color space L*, a*, and b* values, as well as the color strength (K/S) values, were measured at 20 random places using a CHN-Spec CS-650A spectrophotometer (Hangzhou Color Spectrum Technology Company, China). FTIR analysis of grass cloth was carried out using a Nicolet iS5 FT-IR spectroscopy (Thermo Fisher Scientific, USA). The morphology of the raw ramie yarn of grass cloth was investigated with a Phillips SEM scanning electron microscopy (FE-SEM, Germany). The colorfastness towards washing and rubbing of the fabric was tested in accordance with ISO 105-C06:2010 and ISO 105-X12:2016 standards, respectively^30^. Using a multi-fiber strip, colorfastness to washing was determined by measuring neighboring cotton fiber staining and comparing the results to the ISO standard grayscale^31^.

## Results and discussion

### Dyeing of traditional grass cloth

The dyeing performance (E%, F%, and T%) of traditional grass cloth by R2, B194, and O5 and their corresponding standard deviations (Std) of three repeated dyeings are listed in Tables 2, 3, and 4, respectively. In addition, these values were analyzed by the range analysis method, and the results are shown in Tables 5, 6, and 7, respectively. The standard deviations of each of the parameters are close, indicating that it is feasible to repeat the color shade.

**Table 2:** E%, F%, and T% values of R2-dyed samples of traditional grass cloth in the L16 (4^4) orthogonal experimental scheme.

No	Salt (g L^−1^)	Time (min)	Temperature (^o^C)	pH	E% (%)	Std_E%_	F% (%)	Std_F%_	T% (%)	Std_T%_
1	50	30	30	8	49.33	0.41	60.34	0.14	29.77	0.32
2	50	40	40	9	51.03	0.24	69.13	2.81	35.28	1.60
3	50	50	50	10	50.44	1.94	67.98	1.25	34.29	1.90
4	50	60	60	11	45.60	0.16	67.83	0.82	30.93	0.49
5	60	30	40	10	52.55	1.33	71.09	0.15	37.36	1.02
6	60	40	30	11	55.68	0.27	66.13	1.16	36.82	0.47
7	60	50	60	8	53.37	0.61	67.14	0.13	35.83	0.36
8	60	60	50	9	53.28	0.09	67.14	0.40	35.77	0.15
9	70	30	50	11	50.57	0.47	67.18	0.46	33.97	0.52
10	70	40	60	10	54.93	0.38	67.75	1.26	37.22	0.42
11	70	50	30	9	60.91	1.25	62.81	1.50	38.26	1.69
12	70	60	40	8	57.79	1.09	71.18	1.24	41.13	0.06
13	80	30	60	9	56.28	0.17	66.69	0.87	37.53	0.61
14	80	40	50	8	55.75	0.36	66.61	0.51	37.14	0.05
15	80	50	40	11	53.50	1.01	66.62	3.74	35.64	1.35
16	80	60	30	10	64.60	0.13	63.12	0.39	40.78	0.17

**Table 3:** E%, F%, and T% values of B194-dyed samples in the L16 (4^4) orthogonal experimental scheme.

No	Salt (g L^−1^)	Time (min)	Temperature (^o^C)	pH	E% (%)	Std_E%_	F% (%)	Std_F%_	T% (%)	Std_T%_
1	50	30	50	8	34.95	0.52	31.94	0.64	11.16	0.28
2	50	40	60	9	47.27	0.98	46.75	1.30	22.10	1.01
3	50	50	70	10	51.59	1.35	60.48	2.64	31.20	1.59
4	50	60	80	11	57.08	0.57	80.44	0.75	45.92	0.36
5	60	30	60	10	46.58	0.72	50.97	0.72	23.74	0.25
6	60	40	50	11	52.97	3.30	68.29	2.47	36.17	3.41
7	60	50	80	8	54.67	1.54	63.98	0.72	34.98	1.16
8	60	60	70	9	54.74	0.66	50.02	2.58	27.38	1.73
9	70	30	70	11	55.90	2.78	76.60	0.52	42.82	2.21
10	70	40	80	10	55.22	2.02	69.25	4.54	38.24	3.73
11	70	50	50	9	51.28	1.25	37.19	1.83	19.07	1.36
12	70	60	60	8	55.43	0.67	41.07	4.79	22.77	2.88
13	80	30	80	9	53.98	0.53	60.98	2.04	32.92	0.88
14	80	40	70	8	53.81	0.81	46.74	1.35	25.15	0.94
15	80	50	60	11	61.44	2.79	78.77	1.37	48.40	2.95
16	80	60	50	10	58.79	1.66	51.42	3.95	30.23	2.51

**Table 4:** E%, F%, and T% values of O5-dyed samples in the L16 (4^4) orthogonal experimental scheme.

No	Salt (g L^−1^)	Time (min)	Temperature (^o^C)	pH	E% (%)	Std_E%_	F% (%)	Std_F%_	T% (%)	Std_T%_
1	50	30	60	9	49.11	1.19	32.16	1.56	15.79	0.76
2	50	40	70	10	52.93	1.23	57.29	2.59	30.32	2.02
3	50	50	80	11	55.06	2.52	66.38	1.17	36.55	2.11
4	50	60	90	12	44.90	1.25	63.83	2.33	28.66	1.56
5	60	30	70	11	52.75	2.26	64.28	2.83	33.91	2.99
6	60	40	60	12	60.31	2.63	67.71	1.83	40.84	2.53
7	60	50	90	9	52.90	0.90	59.84	0.73	31.66	0.89
8	60	60	80	10	57.11	0.88	65.03	2.41	37.14	1.35
9	70	30	80	12	54.77	1.41	67.66	0.07	37.06	0.97
10	70	40	90	11	55.39	2.24	62.24	3.41	34.47	3.38
11	70	50	60	10	60.48	1.82	43.03	3.03	26.02	2.57
12	70	60	70	9	60.25	0.48	54.78	4.04	33.00	2.67
13	80	30	90	10	53.74	1.04	69.18	1.80	37.18	1.64
14	80	40	80	9	56.89	1.40	52.72	4.30	29.99	3.19
15	80	50	70	12	58.48	1.91	65.32	2.27	38.20	0.05
16	80	60	60	11	67.01	2.67	60.67	1.17	40.65	2.43

**Table 5:** Response table for the means in E%, F%, and T% of R2-dyeings.

Level	Salt (g L^−1^)	Time (min)	Temperature (^o^C)	pH
**E%**				
1	49.10	52.18	57.63	54.06
2	53.72	54.35	53.72	55.38
3	56.05	54.56	52.51	55.63
4	57.53	55.32	52.55	51.34
Delta	8.43	3.13	5.12	4.29
Rank	1	4	2	3
**F%**				
1	32.57	34.66	36.40	35.97
2	36.45	36.61	37.35	36.71
3	37.63	36.01	35.29	37.41
4	37.77	37.15	35.38	34.34
Delta	5.21	2.50	2.06	3.07
Rank	1	3	4	2
**T%**				
1	11.65	13.12	12.69	13.11
2	13.15	13.51	13.50	13.47
3	13.8	12.99	13.28	13.95
4	14.23	13.22	13.38	12.31
Delta	2.58	0.52	0.81	1.65
Rank	1	4	3	2

**Table 6:** Response table for the means in E%, F%, and T% of B194-dyeings.

Level	Salt (g L^−1^)	Time (min)	Temperature (^o^C)	pH
**E%**				
1	47.72	47.85	49.50	49.72
2	52.24	52.32	52.68	51.82
3	54.46	54.75	54.01	53.04
4	57.00	56.51	55.24	56.85
Delta	9.28	8.66	5.74	7.13
Rank	1	2	4	3
**F%**				
1	54.90	55.12	47.21	45.93
2	58.32	57.76	54.39	48.73
3	56.03	60.10	58.46	58.03
4	59.48	55.74	68.66	76.03
Delta	4.58	4.98	21.45	30.09
Rank	4	3	2	1
**T%**				
1	27.59	27.66	24.16	23.51
2	30.57	30.42	29.25	25.37
3	30.72	33.41	31.64	30.85
4	34.17	31.57	38.01	43.33
Delta	6.58	5.75	13.85	19.81
Rank	3	4	2	1

**Table 7:** Response table for the means in E%, F%, and T% of O5-dyeings.

Level	Salt (g L^−1^)	Time (min)	Temperature (^o^C)	pH
**E%**				
1	50.5	52.59	59.23	54.79
2	55.77	56.38	56.1	56.06
3	57.72	56.73	55.96	57.55
4	59.03	57.32	51.73	54.62
Delta	8.53	4.72	7.5	2.94
Rank	1	3	2	4
**F%**				
1	54.91	58.32	50.89	49.88
2	64.22	59.99	60.42	58.63
3	56.93	58.64	62.95	63.39
4	61.97	61.08	63.77	66.13
Delta	9.3	2.76	12.88	16.25
Rank	3	4	2	1
**T%**				
1	27.83	30.98	30.83	27.61
2	35.88	33.91	33.86	32.67
3	32.64	33.11	35.18	36.4
4	36.51	34.86	32.99	36.19
Delta	8.67	3.88	4.36	8.78
Rank	2	4	3	1

For dye exhaustion, the E% values of R2 were in a range of 45.60% to 64.60%, B194 were in the range of 34.95% to 61.44%, and O5 were in the range 44.90% to 67.01%, which show range spans of 19.00%, 26.49%, and 22.11%, respectively. The highest difference for B194 demonstrates that the dyeing conditions more sensitively affect E% than the other two dyes. Moreover, it is worth noting that the salt factor plays the most important influence relative to E% among the three dyes, since it was ranked at first position in Table 5 (E%), Table 6 (E%), and Table 7 (E%). In addition, E% increased with increasing salt concentration from 50 to 80 g L^−1^ (salt response on E% in Tables 5, 6, and 7). In the reactive dyeing of grass cloth, dye exhaustion includes not only physical adsorption, but also chemical absorption. In physical adsorption, salt addition promotes dye exhaustion because it reduces the repulsive forces between the anionic reactive dye and anionic cellulosic and gummy materials. In chemical absorption, the covalent reaction (i.e. dye fixation) breaks the physical adsorption and further promotes the dye’s physical adsorption. Thus, chemical absorption is beneficial for the E% value^32^.

**Figure 3:**
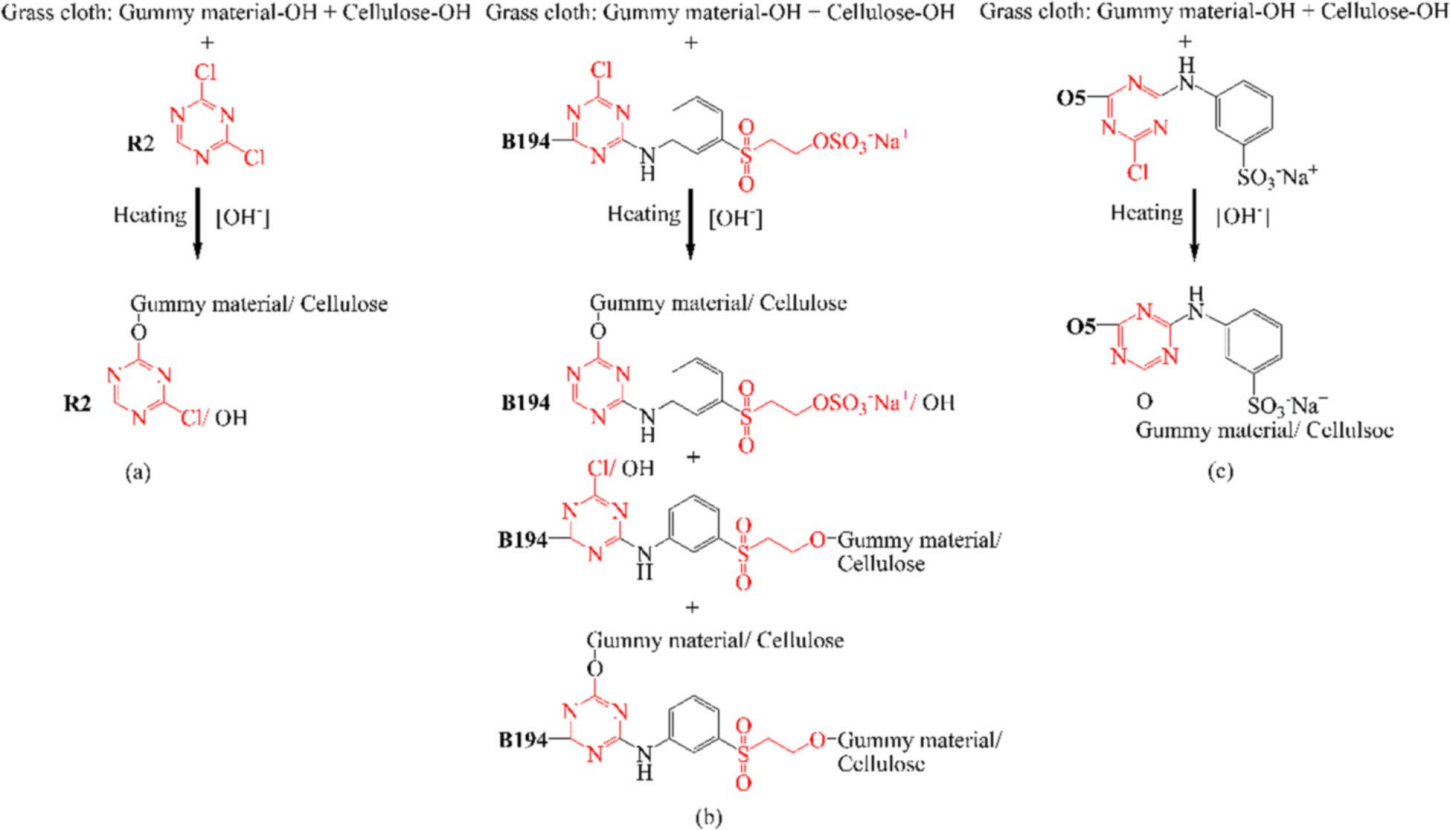
Interaction of grass cloth with the functional sites of R2 (**a**), B194 (**b**), and O5 (**c**).

In dye fixation, the F% values of dyeings with R2 were in a range of 60.34% to 71.18%, B194 were in the range of 31.94% to 80.44%, and O5 were in the range of 32.16% to 69.18%, which yield spans of 10.84%, 48.50%, and 37.02%, respectively. The highest difference value for B194 suggests that dyeing conditions more sensitively affect F% than the other two dyes. Figure 1 shows that R2 has one dichlorotriazinyl (DCT) group, which is more reactive compared to the vinyl sulfone (VS) in B194 and monochlorotriazinyl (MCT) groups in O5 and B194^32^. This means that R2 covalently bonds more easily with the traditional grass cloth, but it also more easily hydrolyzes during dyeing, which contrasts with B194 and O5. During alkaline dyeing, the hydroxyl groups of cellulose and the gummy material (cellulose–OH and gummy material–OH) were transferred to the oxygen anions of cellulose and gummy material (cellulose–O^−^ and gummy material–O^−^, Fig. 3), respectively; meanwhile, the reactive groups were activated to excited situations^33^. Thus, raising the pH of the dyebath to alkaline expedited the covalent reaction. Also, a high fixing temperature benefited the covalent reaction since it is endothermic^34^. However, hydrolysis of the reactive group occurred simultaneously. In other words, the more reactive group quickly reacted with the grass cloth and was more easily hydrolyzed.

At alkaline conditions, the DCT group of R2 was first hydrolyzed^35^ to a MCT group, resulting in weakened reactive properties. Subsequently, the MCT group was possibly further hydrolyzed^36^ to a completely hydrolyzed form, which began to lose its reactivity. Since the DCT group is more reactive (i.e. easily reacted with the grass cloth in mild alkaline conditions), the fixation conditions of dyeing (mainly pH of the dyebath and fixing temperature) were less sensitive to R2 when compared to 94 and O5. Thus, dyebath pH and fixing temperature were ranked second and fourth in R2 fixation. However, each factor was ranked first and second, respectively, for B194 and O5, as shown in the pH response on F% in Tables 6 and 7. This indicates that the less reactive MCT and VS groups are more sensitive to pH changes and fixing temperature within that range. In dyeing with B194 and O5, the F% values increase with increasing pH, but when dyeing with R2, the F% values increase with pH increase to 10, and then decrease at pH 11, which is possibly ascribed to R2 hydrolysis.

In dye fixation, T% is dependent on its E% and F%, which refers to the utilization rate of dye mass usage. The T% values of dyeing with R2 were in a range of 29.77% to 41.13%, B194 were in the range of 11.16% to 48.40%, and O5 were in the range of 15.79% to 40.84%, which show range differences of 11.37%, 37.23%, and 25.04%, respectively. The highest T%, 48.40%, was achieved in the B194 under dyeing conditions of 80 g L^−1^ of NaCl salt, 50 min of fixing time, 60 °C fixing temperature, and pH 11 (Table 3, No. 15), accompanied by 61.44% of E% and 78.77% of F%. Although the F% (78.77%) was not the highest value (80.44%), its highest E% (61.44%) made a huge contribution to the T% value. Therefore, the influence of dyeing factors on T% was complicated^20^, and it is worth closely analyzing with the Taguchi analysis method.

### Taguchi analysis of T% of B194 dyeings

The color shade of grass cloth is dependent on the total dye mass in the dyed grass cloth, which is contributed by its dye exhaustion and dye fixation. When the masses of substance and initial dye are fixed, the T% expresses the dye utilization rate and is then used to characterize the color shade in comparison to the dyeing performance among the different dyeing conditions. In addition, the highest T% among these three dyes was presented in the B194 dyeing. Therefore, T% of B194 dyeing was selected to do the Taguchi analysis.

In Taguchi analysis, the S/N ratio is used to assess how the actual value differs from the intended one, in which the signal represents the desirable values and noise denotes the undesirable values. There are three types of S/N ratios available, and “the-larger-the-better” (Eq. 4)^37^ was selected according to the objectives of this study (higher fixation rate).

4 $$\text{S/N}{=}- \text{10 log}\left(\frac{1}{{\text{n}}}{\sum }_{{\text{i}}= \text{1} }^{\text{n}}\frac{1}{{\text{y}}_{\text{i}}^{2}}\right)$$

where y_i_ represents the ith experiment in the orthogonal array design, and n indicates the total number of experimental runs. The average S/N values were calculated at the four-level for the four factors, and the response results are presented in Table 8. From this analysis, the factor with the greatest mean S/N ratio is assumed to have an ideal value. In certain ways, delta characteristics were also measured by subtracting the greatest and lowest average S/N ratio values, which are necessary in determining the most influential factors^27^. After that, the values are allocated according to a ranking system, which means that rank 1 indicates the greatest value, followed by rank 2 and rank 3 and so on. It was evident that solution pH revealed the most influential factor with a maximum delta value of 5.94. This was followed by temperature (4.69), salt (2.67), and time (2.15), as noted by the rankings of 2, 3, and 4, respectively.

**Table 8:** Response table for S/N ratios in T% of B194 dyeings.

Level	Salt (g L^−1^)	Time (min)	Temperature (^o^C)	pH
1	27.74	27.86	26.83	26.75
2	29.58	29.43	28.81	27.90
3	29.26	30.02	29.82	29.66
4	30.42	29.69	31.53	32.68
Delta	2.67	2.15	4.69	5.94
Rank	3	4	2	1

**Figure 4:**
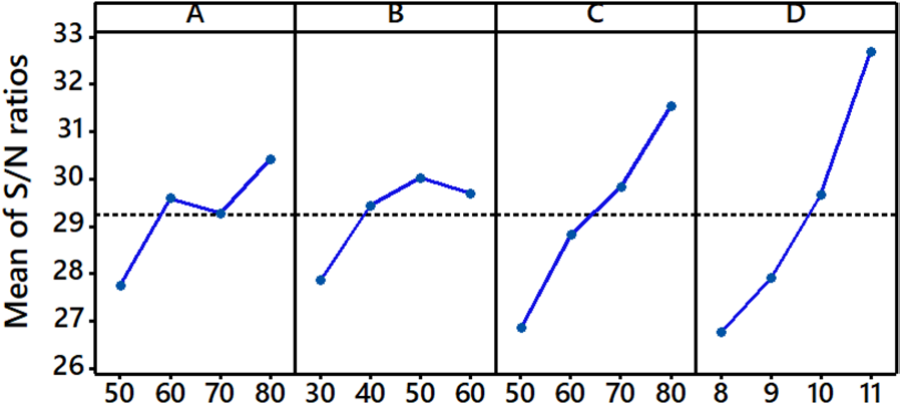
Main effects plot for S/N ratios (data means) in T% of B194-dyeings.

Additionally, Fig. 4 represents the main effects plot of the process parameters for S/N ratios (data means) in T% of B194-dyeing. By comparing the values of the individual process parameters to a solid line, the productivity of the various process parameters is shown. If a particular process parameter is near the solid line, it indicates that the process has a minor influence on the dyeing process. On the other hand, the dyeing process is primarily affected by a parameter with a higher slope. Consequently, fixation temperature (C) and pH (D) were shown to have a statistically significant influence on dyeability among the parameters evaluated, whereas dying fixation time (B) and salt concentration (A) exhibited a very modest impact. Considering this, the optimal conditions are designated as A3B3C4D4, which led to the highest possible T% when using the Taguchi technique, as the highest possible T% represents the most efficient dyeing performance.

**Figure 5:**
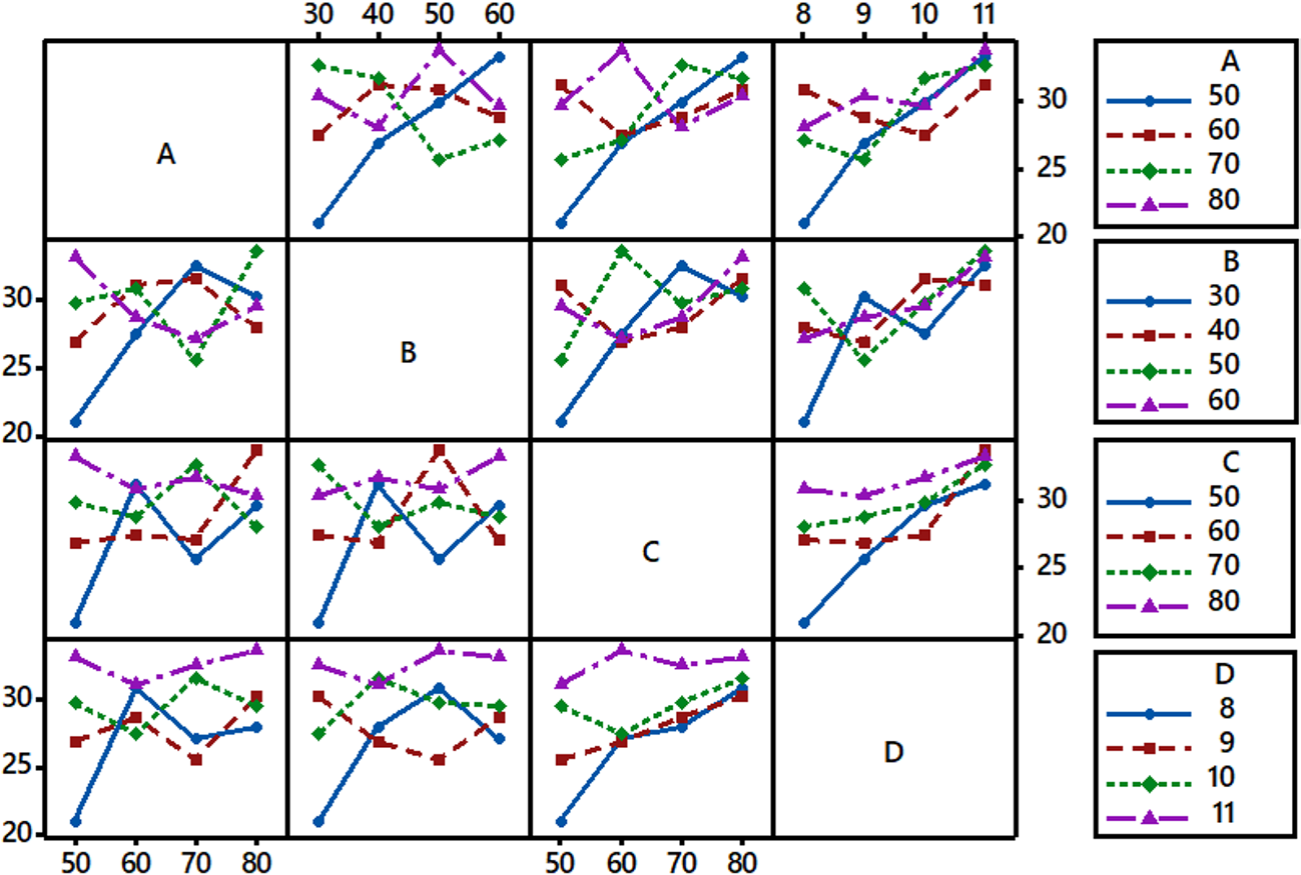
Full interaction plot for S/N ratios (T%).

To determine the interaction behavior between the levels of process parameters, an assessment of the interaction graph is essential. Parallelism and non-parallelism are the two sorts of interaction behaviors that might be observed. The plot's non-parallel and parallel lines may be used to identify the interaction effects of the input parameters. Non-parallel lines show substantial interdependence between parameters, whereas parallel lines show modest interdependence^38,39^. From the interaction plot as shown in Fig. 5, there is substantial interaction between the three variables of salt (A), fixation time (B), and fixation temperature (C), which may be seen as non-parallel lines (three lines intersect with each other). On the other hand, pH (D) was discovered to have essentially parallel lines, showing that their levels were not as dependent on each other as previously thought. Since interaction plots are excellent at examining process factors, this research shows that the chosen parameters had an enormous impact on the reactive dyeing of grass cloth.

It is possible to utilize the S/N ratio to calculate the optimal level of each component. Nevertheless, it is unable to offer information on the experiment's most important element. ANOVA tests may be used to determine the relative importance of different variables in an optimized model based on an orthogonal experimental design. In the beginning, it was intended to have a distribution of measurements, a sum of squares (SS), and then to divide it by SS factors derived from the trial findings themselves. For Fischer's test (F-value), the ratio of MS values, which indicates the model's most critical component, was used. Significant factors are those that show a p-value less than 0.05; this is the threshold at which they are regarded to be significant.^29,40^. Table 9 displays the ANOVA findings for T%. Dyebath pH was shown to have a considerable effect on T%, with a highest F-value of 14.49, a p-value of 0.02 (significant), and an overall contribution percentage as high as 50%. There were also significant (p-value 0.05) results for other variables, such as the fixation temperature and the percentage contribution of 29.25%. Furthermore, the fixation time and salt concentration contribution percentages were 6.93% and 9.48%, with the latter two contributing the least rendered non-significant.

**Table 9:** ANOVA for S/N ratios of total dye fixation efficiency (T%).

Source	DF	SS	MS	F	p-value	Remarks	P (%)
A	3	14.97	4.98	2.70	0.21	Not significant	9.48
B	3	10.94	3.64	1.98	0.29	Not significant	6.93
C	3	46.17	15.38	8.34	0.05	Significant	29.25
D	3	80.22	26.74	14.49	0.02	Significant	50.83
Residual Error	3	5.54	1.84				3.51
Total	15	157.83					

**Figure 6:**
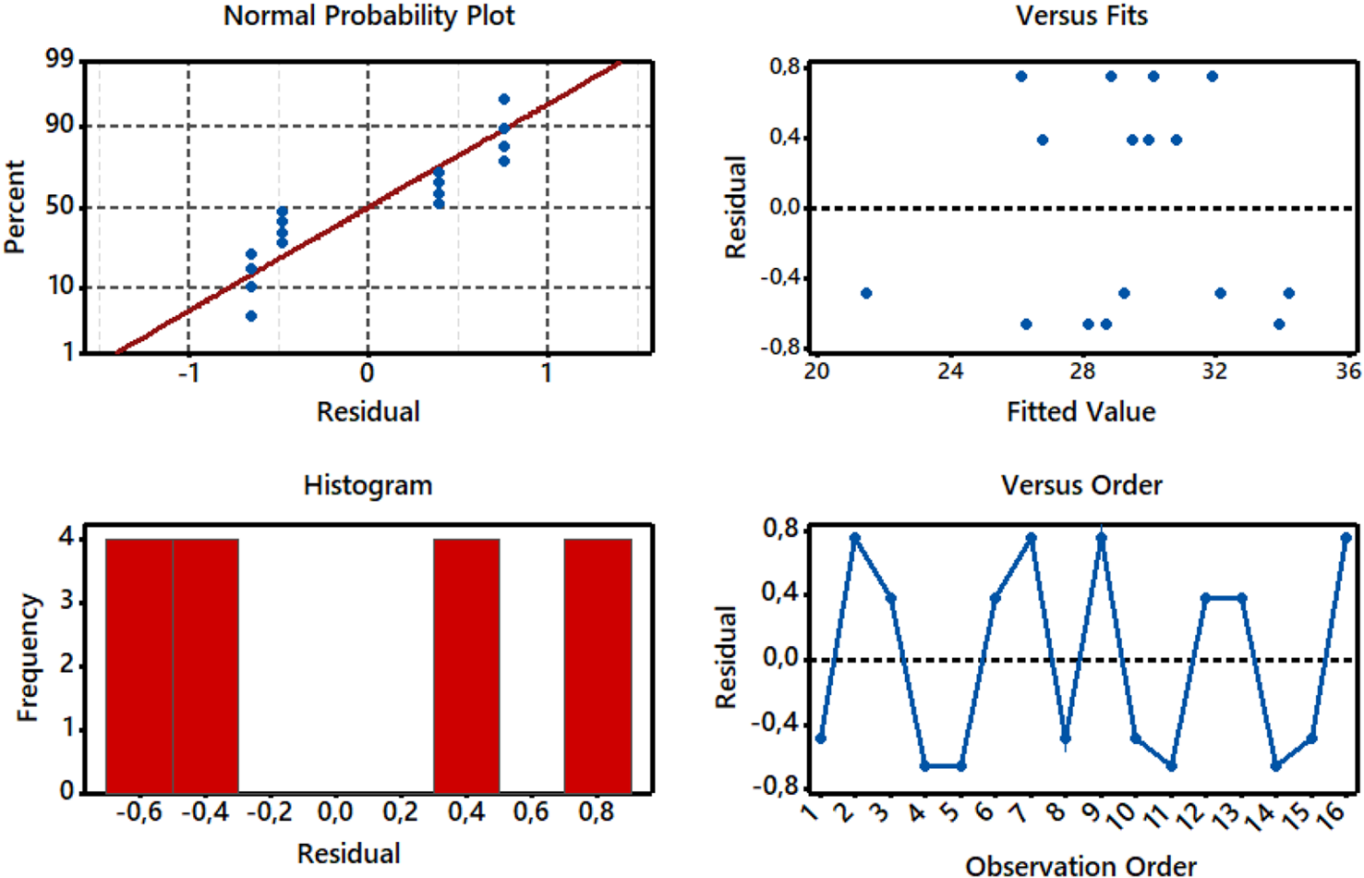
Residual plots for S/N ratios on normal probability, versus fits, histogram, and versus order.

The residual plots (normal probability plot, versus fits, histogram and versus order) for S/N ratios of total dye fixation efficiency (T%) are shown in Fig. 6. The obtained normal probability plot exhibits a similar pattern with most spots on or near the line, indicating that residuals are normally distributed throughout the dyeing process. The residual vs fitted values plot is employed to assess whether the output findings are affected by the specified parameters. It reveals that lower spots are oriented progressively horizontally, whereas upper spots are oriented more arbitrarily towards the residual lines (zero value), demonstrating that residuals have a constant variance relevance^41,42^. As seen from the histogram bar chart, at very few observations, residuals have a variance point. Finally, the residuals versus order study indicates that observed residuals are randomly distributed towards the zero lines, underscoring that residuals are strongly dependent on dyeing operation.

**Figure 7:**
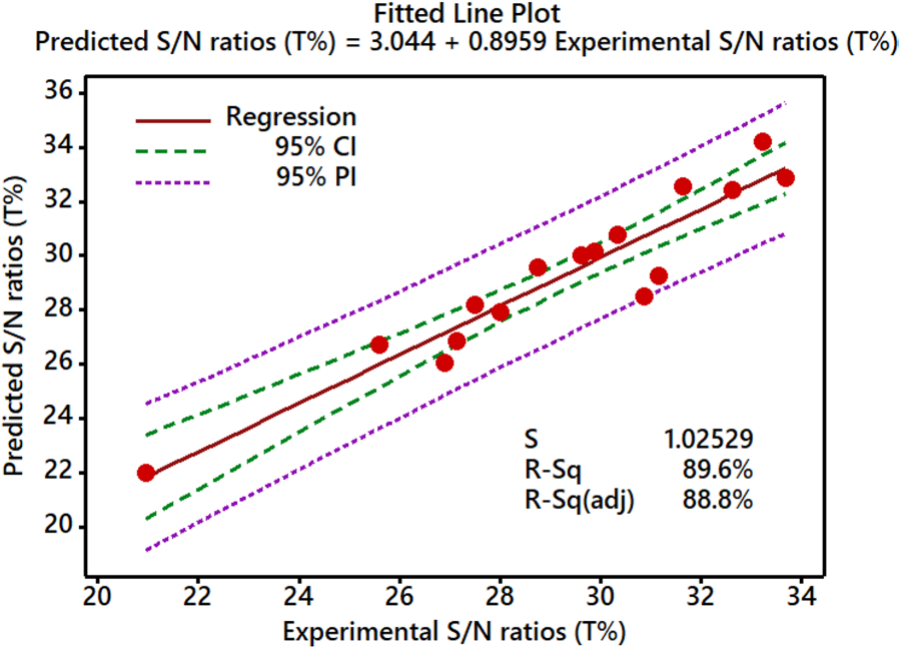
Fitted lines for the experimental S/N ratios (T%) and predicted S/N ratios (T%).

With the regression equation, it is possible to correctly compute and compare projected values based on experimental circumstances^20^. For the T% of reactive dye, the fitted plots of expected vs experimental responses are shown in Fig. 7. An R^2^ value of 89.6%, along with an adjusted R^2^ of 98.9%, clearly shows a good match between the experimental and projected T%. The values, on the other hand, are strongly aligned. The model’s capacity to accurately forecast the answer is evident when the variation around the mean of the responses is smaller. This study’s P-value is 0.000, and the Pearson correlation coefficient between the anticipated and actual T% was 0.8959. This shows that the expected and actual fixation rates are strongly correlated^43^.

In the Taguchi approach, the confirmation test is necessary to clarify the acquired results, and it is highly advised for statistical methods. The major goal of this study is to verify the validity of the tests and responses^44^. The findings of the confirmation trials are shown in Table 10. Once optimal conditions have been deduced, the next step is to check that the process has been optimized. The expected values were established with the use of software. As a result, the experiment was carried out using the optimal settings, and it was discovered that a sufficient improvement in the S/N ratio had been achieved. The T% was improved (with an increase in the S/N ratio of 0.82), which was the primary goal of this research. These findings revealed that improved performance could be achieved using a consistent, statistical experimental design.

**Table 10:** Results of the confirmation experiment.

Conditions	Initial parameters	Prediction	Confirmation experiment
Level	A4B3C2D4	A4B3C4D4	A4B3C4D4
T%	48.40	56.62	53.18
S/N	33.69	36.90	34.51
Improvement in the S/N ratio		0.82	

### Characterization

**Figure 8:**
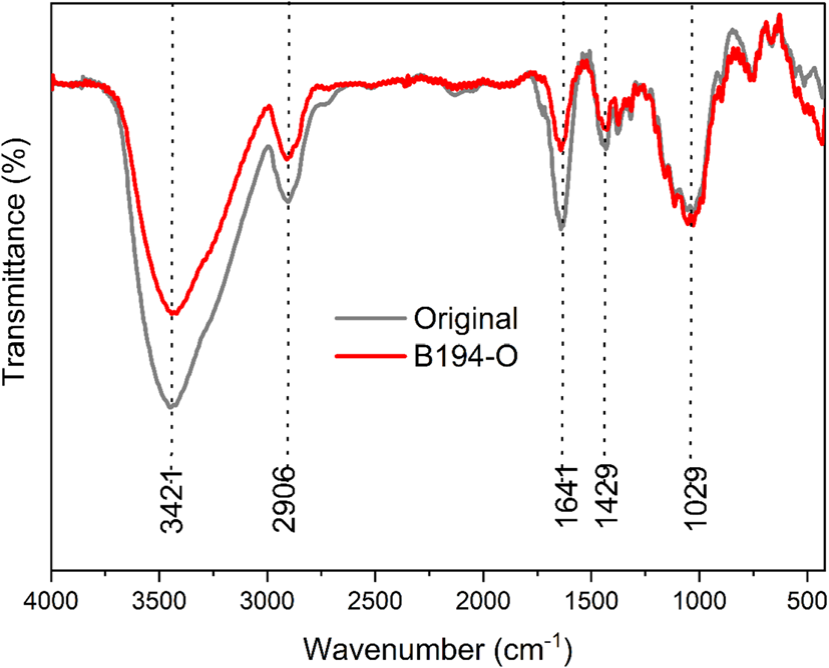
FTIR spectra of original and optimized conditions dyed samples by B194.

**Figure 9:**
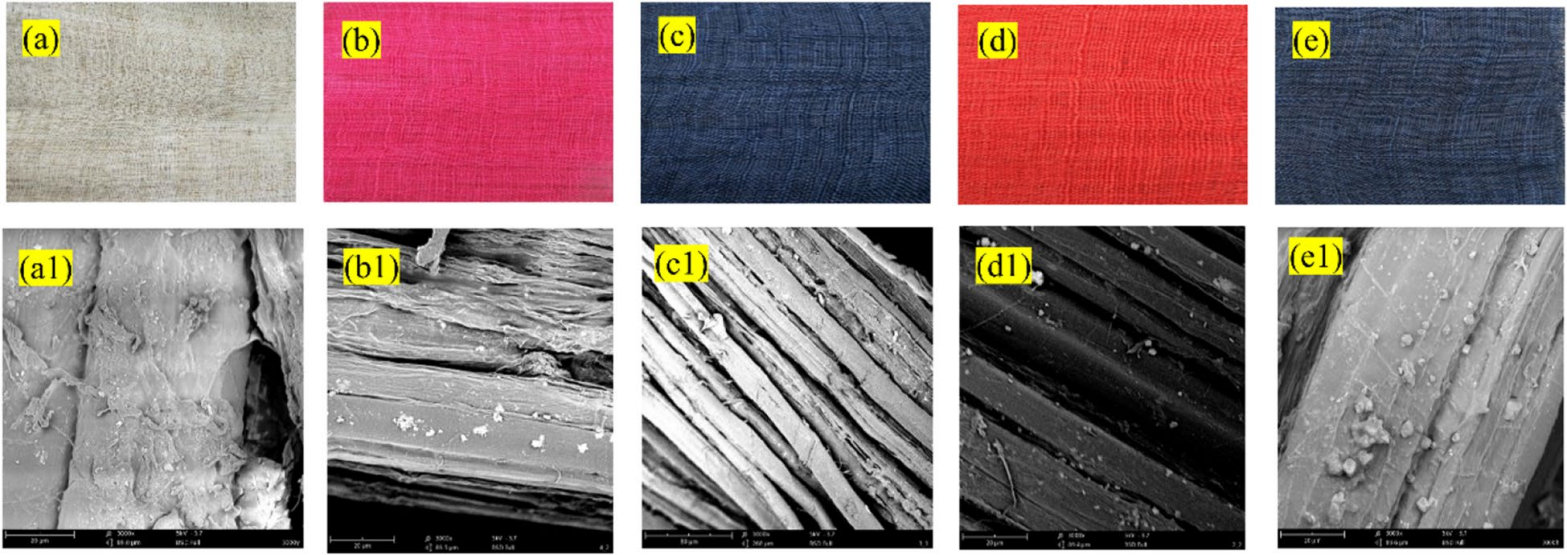
Pictures of (**a,a1**) original and (**b,b1**) R2, (**c,c1**) B194, (**d,d1**) O5, (**e,e1**) B194-O dyed traditional grass cloth and with their SEM images, respectively.

FTIR analysis was used to determine the diversity of functional groups in the original grass cloth and optimized conditioned dyed sample by B194 (B194-O). As shown in Fig. 8, a dominant characteristic peak appeared at 3421 cm^−1^ due to the –OH stretching vibration of hydroxyl and phenolic groups. Next, a peak around 2906 cm^−1^ could be ascribed to the stretching vibration of –CH groups^45^. Peaks at 1641 cm^−1^ corresponded to the C=O acetyl group, at 1429 cm^−1^ was assigned to the C–H bending vibration, and near 1028 cm^−1^ is due to the stretching vibration of the C–O–C bond^46–48^. Compared with the original grass cloth, the main characteristic peaks were still present, suggesting that non-cellulosic compounds were removed during the dyeing process. Some gummy materials were still noticed on the dyed fiber surface, as correlated with the SEM images, as shown in Fig. 9e1.

The photographs and SEM images of original traditional grass cloth and after soaped grass cloth dyed samples, including R2-dyed (Table 2, No. 12), B194-dyed (Table 3, No. 15), O5-dyed (Table 4, No. 6), and optimized conditioned B194-dyed (B194-O) are exhibited in Fig. 9. The dyed samples showed satisfactory color shades, demonstrating that traditional reactive dyeing procedures could achieve effective coloration of grass cloth. Moreover, the dyeing procedures were not harmful to the presence of gummy materials, because the gummy materials existed in the grass cloth before and after the dyeing procedures, as shown in the SEM images.

Furthermore, the L*, a*, and b* values, K/S values, and colorfastness to washing and rubbing of the dyed samples are listed in Table 11. The L* value of the B194-dyed sample was less than the other two dyes, which means the B194-dyed sample was darker. The B194-O sample appeared darker since its L* value was 18.8, somewhat lower than that of the B194 sample. In addition, the K/S value of the B194-O sample was 22.89, slightly higher than that of the B194 dyed sample, which showed that the grass cloth dyed using B194 at optimized conditions obtained the best color strength. The colorfastness to washing and rubbing of all dyed samples were at a high level, Grade 4 or higher, which certified that the colorfastness of the dyed grass cloth was satisfactory.

**Table 11:** Chromatic values and colorfastness of dyed grass clothes.

Dyed sample	Chromatic values				Wash fastness (Grade)		Rubbing fastness (Grade)	
	L*	a*	b*	K/S	Staining	Fading	Dry	Wet
R2	36.2	44.9	3.2	14.73	4	5	4–5	4
B194	19.6	−1.1	−9.4	22.23	4	5	4–5	4
O5	38.5	41.7	26.8	13.71	4	5	4–5	4
B194-O	18.8	−0.8	−8.2	22.89	4	5	4–5	4

## Conclusions

In this study, the dyeing behavior of sustainable grass cloth fiber was thoroughly investigated using three types of commercial reactive dyes, and it was determined that the B194 dye achieved the highest total fixation rate. After that, the implementation of the Taguchi design assisted in obtaining the optimal dyeing conditions (A4B3C4D4) with a salt concentration of 80 g L^−1^, fixation time of 50 min, fixation temperature of 80 °C, and solution pH 11. Then, ANOVA analysis revealed that solution pH was the primary contributor with an amount of 50%. Fitted models of experimental vs predicted T% showed that they are strongly correlated (P-value is 0.000). T% was examined under optimal conditions, which showed higher (53.18%) value than at initial conditions (48.40%), which can be correlated to the photograph of the B194-dyed sample. Additionally, color coordinates and fastness properties were satisfactory. However, the current work could only deal with reactive dye, and the total fixation rate was also not so high. Accordingly, the study on sustainable dyeing of grass cloth should be carried out by applying natural dyes and various anhydrous methods such as microemulsion and supercritical carbon dioxide. At the same time, the use of a cationic fixing agent might help enhance the total fixation rate. Overall, these issues are essential aspects of textile dyeing industries, which must be sorted out to broaden their future commercial uses.

## Data Availability

The datasets generated during the current study are available from the corresponding author on reasonable request (Prof. Yingjie Cai, Y. Cai).

## Code availability

No code was attempted or used during the current manuscript.
